# Baicalin Attenuates Panton–Valentine Leukocidin (PVL)-Induced Cytoskeleton Rearrangement via Regulating the RhoA/ROCK/LIMK and PI3K/AKT/GSK-3β Pathways in Bovine Mammary Epithelial Cells

**DOI:** 10.3390/ijms241914520

**Published:** 2023-09-25

**Authors:** Jiangliu Yang, Zhenzhen Hai, Ling Hou, Yang Liu, Dongtao Zhang, Xuezhang Zhou

**Affiliations:** Key Laboratory of the Ministry of Education for the Conservation and Utilization of Special Biological Resources of Western China, Ningxia University, Yinchuan 750021, China; nptt2005@163.com (J.Y.); zzh19980507@163.com (Z.H.);

**Keywords:** rPVL, cytoskeleton rearrangement, baicalin, RhoA/ROCK/LIMK, PI3K/AKT/GSK-3β, bovine mastitis

## Abstract

Pore-forming toxins (PFTs) exert physiological effects by rearrangement of the host cell cytoskeleton. *Staphylococcus aureus*-secreted PFTs play an important role in bovine mastitis. In the study, we examined the effects of recombinant Panton–Valentine leukocidin (rPVL) on cytoskeleton rearrangement, and identified the signaling pathways involved in regulating the process in bovine mammary epithelial cells (BMECs) in vitro. Meanwhile, the underlying regulatory mechanism of baicalin for this process was investigated. The results showed that *S*. *aureus* induced cytoskeleton rearrangement in BMECs mainly through PVL. *S*. *aureus* and rPVL caused alterations in the cell morphology and layer integrity due to microfilament and microtubule rearrangement and focal contact inability. rPVL strongly induced the phosphorylation of cofilin at Ser3 mediating by the activation of the RhoA/ROCK/LIMK pathway, and resulted in the activation of loss of actin stress fibers, or the hyperphosphorylation of Tau at Ser396 inducing by the inhibition of the PI3K/AKT/GSK-3β pathways, and decreased the microtubule assembly. Baicalin significantly attenuated rPVL-stimulated cytoskeleton rearrangement in BMECs. Baicalin inhibited cofilin phosphorylation or Tau hyperphosphorylation via regulating the activation of RhoA/ROCK/LIMK and PI3K/AKT/GSK-3β signaling pathways. These findings provide new insights into the pathogenesis and potential treatment in *S*. *aureus* causing bovine mastitis.

## 1. Introduction

*S*. *aureus* is a facultative intracellular bacterium causing a variety of severe diseases. In dairy cows, *S*. *aureus* causes clinical mastitis and subclinical mastitis with great economic loss and serious animal health problems [1]. Toxins play a preeminent role in *S*. *aureus* virulence, and mostly aim to lysate cells and evade elimination by host defenses [2]. The pore-forming toxins (PFTs) secreted by *S*. *aureus* are a class of exotoxins including α-hemolysin (Hla), β-hemolysin (Hlb), leukocidin, phenolsoluble modulins (PSMs), and epidermal cell differentiation inhibitor (EDIN) [3,4]. *S*. *aureus* PFTs such as Hla, Hlb, and leukocidin LukMF’ are intimately involved in intramammary infections by altering the plasma membrane permeability, leading to cell death [5,6,7,8]. In vitro studies showed that *S*. *aureus* infection of bovine mammary epithelial cells (BMECs) involves actin cytoskeleton rearrangement [9,10]. Hla, PSMs, and EDIN can also exert physiological effects by rearrangement of the host cell cytoskeleton [11,12,13]. However, the role of cytoskeletal disruption due to PFTs in *S*. *aureus*-induced bovine mastitis remains unknown.

The mammalian cell cytoskeleton is crucial for many diverse cellular functions, such as cell structure and motility, cell division, and phagocytosis [14,15]. The abnormal assembly of the cytoskeleton of BMECs causes the inhibition of bacterial adherence and invasion [16,17,18]. Mammalian cells respond to toxins by rearranging the cytoskeleton through intracellular signaling systems. As primary regulators of the actin cytoskeleton, Rho GTPases are important targets for bacterial protein toxins. Rho GTPases, including Rho, Rac, and Cdc42, are activated/inactivated by toxins and regulated downstream effectors of ROCK, thus inducing the phosphorylation of cofilin (an actin-severing protein) at ser3, and resulting in actin polymerization or depolymerization [19,20]. Previous studies showed that *S*. *aureus* infection induced BMECs actin cytoskeleton rearrangement through Rho GTPase (RhoA) regulated pathway modulation [9]. Hence, it may be that PFTs are involved in the destruction of BMECs by activating the RhoA pathway. The PI3K/AKT signaling pathway plays an important role in a variety of cellular processes, the activation of PI3K/AKT signaling influences cytoskeletal changes in various cells types [21]. The PI3K/AKT signaling pathway dysfunction causes Tau hyperphosphorylation, and decreases its microtubule assembly and stabilization activity [22,23]. Pathogens such as *K*. *pneumoniae* [24], *L*. *monocytogenes* [25], *E*. *coli* K1 [26], and *P*. *aeruginosa* [27] have been shown to activate PI3K/Akt signaling to promote internalization to cells.

Panton-Valentine leukocidin (PVL) is another PFT, and it has been associated with primary *S*. *aureus* skin infections and pneumonia [28]. Recently, methicillin-resistant *S*. *aureus* (MRSA) has been shown to cause bovine mastitis (prevalence is 4.30%) [29], and MRSA with the *pvl* gene has been increasingly detected in the milk of bovine mastitis worldwide [30,31]. Our previous study showed that both PVL-produced *S*. *aureus* and recombinant PVL (rPVL) induce apoptosis and necrosis in BMECs in vitro [32]. As the main bioactive components extracted from Chinese herbal medicine *Scutellaria radix*, baicalin displays various pharmacological activities, including antitumor, antimicrobial, and antioxidant activities, and has wide clinical applications [33]. We have shown that baicalin can attenuate rPVL-induced BMECs damage [32]. Given that *S*. *aureus* PFTs-induced actomyosin rearrangements are closely linked to cell functions such as cell migration or apoptosis induction, and the actin cytoskeleton regulation role of baicalin [34,35], we assessed the BMECs cytoskeletal responses to rPVL, and investigated the effects and molecular mechanisms of baicalin in this pathological process. The study may provide a reference for the pathogenesis and potential treatment of *S*. *aureus* -induced mastitis in dairy cows.

## 2. Results

### 2.1. rPVL Caused the Rearrangement of the Microfilaments and Microtubules in BMECs

**Figure 1:**
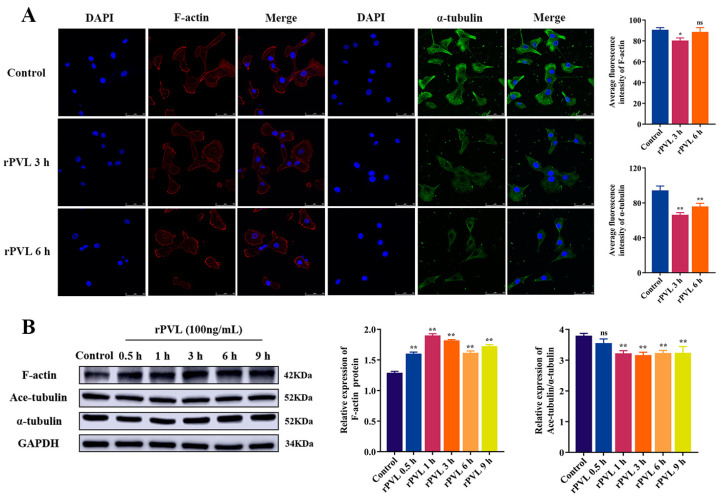
Rearrangement of the microfilaments and microtubules in BMECs treated with rPVL. (**A**) Damage on the microfilaments and microtubules morphology in the rPVL-stimulated BMECs as observed by immunofluorescence. Labeled with Rhodamine–Phalloidin (red), anti-α-tubulin antibody (green) and DAPI (blue). Scale bars are 50 μm in all figures. (**B**) Effects of rPVL on the abundance of F-actin, Ace-tubulin, and α-tubulin in BMECs. Western blot was used to determine the relative levels of F-actin, Ace-tubulin and α-tubulin; GAPDH was used as a control. rPVL was used at a concentration of 100 ng/mL. Data are expressed as mean ± standard deviation of three independent experiments, * 0.01 < *p* < 0.05, ** *p* < 0.01 (one-way ANOVA with Dunnett’s multiple comparison tests), ns: not significant.

We proved that PVL-treated BMECs showed cytoplasmic vacuolization, nuclei chromatin condensation, and vacuolated DNA fragmentation [32]. To further explore the damaging effects of rPVL on BMECs, the effects of rPVL on the cytoskeleton of BMECs were investigated. Immunofluorescence staining revealed that BMECs in the control group exhibited an epithelioid shape with polygonal extensions; numerous cells actin stress fibers (red fluorescence) and microtubules (green fluorescence) passed through the entire cell width with a scattered arrangement; focal contact-rich lamellipodia distributed at the cell layer border. In contrast, the treatment of BMECs with 100 ng/mL rPVL showed cell rounding and dissociation, cytoplasmic vacuolization, and retraction of plasma membrane protrusions. Exposure of rPVL for 3 h obviously diminished the levels of actin filaments and polymerized microtubules compared to control cells (with decreased fluorescence intensity, *p* < 0.05, Figure 1A). Moreover, strong peripheral actin filaments were accumulated in the marginal zone of the cytoplasm at 6 h (Figure 1A). Western blot showed that 100 ng/mL rPVL-induced F-actin abundance was significantly increased (*p* < 0.01, Figure 1B), and the ratio of Ace-tubulin/α-tubulin was decreased after treatment (*p* < 0.05, Figure 1B). These results suggest that rPVL reduces the stability of microfilaments and microtubules, as well as improper realignment of the cytoskeleton in BMECs.

### 2.2. S. aureus Infection-Induced Microfilament and Microtubule Rearrangement Mainly through PVL

**Figure 2:**
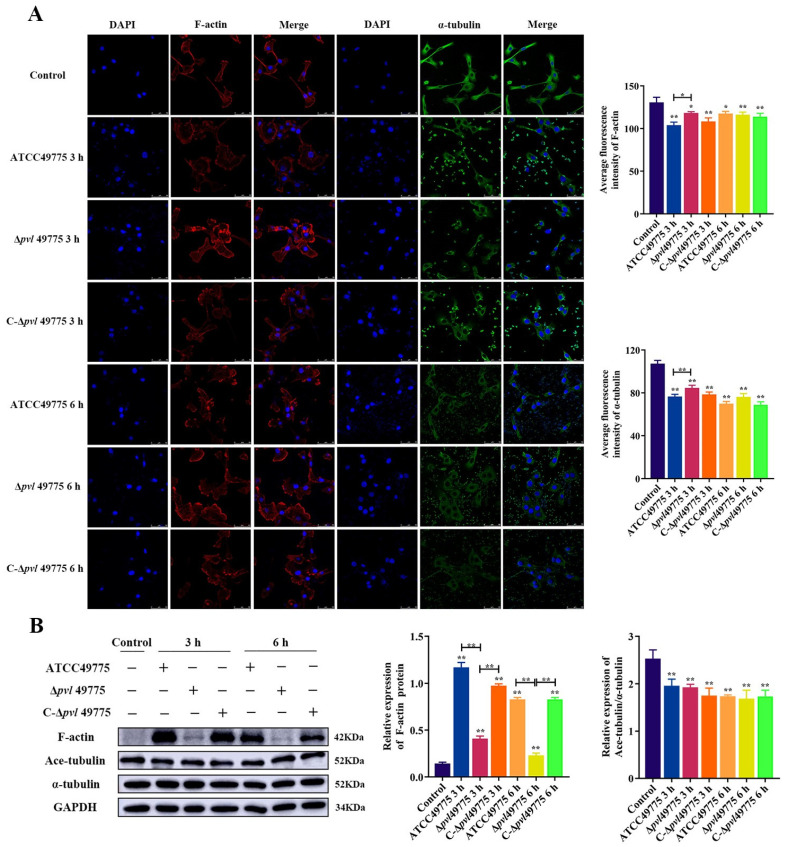
Rearrangement of the microfilaments and microtubules in BMECs treated with *S. aureus*. (**A**) Damage caused by *S. aureus* on the morphology of microfilaments and microtubules in BMECs by immunofluorescence. Labeled with Rhodamine–Phalloidin (red), anti-α-tubulin antibody (green), and DAPI (blue). Scale bars are 50 μm in all figures. (**B**) Effects of *S. aureus* on the relative abundance of F-actin, Ace-tubulin, and α-tubulin in BMECs. Western blot was used to determine the expression levels of F-actin, Ace-tubulin, and α-tubulin; GAPDH was used as a control. The MOI of bacteria counted in the experimental group was 30. Data are expressed as mean ± standard deviation of three independent experiments. * 0.01 < *p* < 0.05, ** *p* < 0.01 compared to the control, unless indicated with brackets (one-way ANOVA with Tukey’s multiple comparison tests).

We observed that 100 ng/mL rPVL induced cytoskeleton damage in BMECs, similar to what was observed for intact *S*. *aureus* ATCC49775 (Figure 2A), suggesting that PVL secreted by *S*. *aureus* is responsible for the induction of cytoskeleton damage by this bacterium. To confirm this, we investigated a *pvl*-deficient *S*. *aureus* strain Δ*pvl* 49775; its complemented mutant strain C-Δ*pvl*49775 induced cytoskeleton damage; we found that microfilament and microtubule remodeling induced by *S*. *aureus* Δ*pvl* 49775 was significantly attenuated compared with that in *S*. *aureus* ATCC49775 at 3 h (with increased fluorescence intensity, *p* < 0.05, Figure 2A), cell morphology partially restored. It was shown that *S*. *aureus* C-Δ*pvl* 49775 restored the ability to induce microfilament and microtubule remodeling. Thus, PVL is responsible for the ability of *S*. *aureus* to induce cytoskeleton remodeling in BMECs. We also examined the abundance of protein make-up microfilaments and microtubules infected with *S*. *aureus*. Compared with *S*. *aureus* 49775 and C-Δ*pvl* 49775, Δ*pvl* 49775-induced abundance of F-actin was significantly downregulated (*p* < 0.01, Figure 2B). No difference in the Ace-tubulin/α-tubulin ratio between Δ*pvl* 49775, *S*. *aureus* 49775, and C-Δ*pvl* 49775 (Figure 2B) was observed.

### 2.3. rPVL Activated the RhoA/ROCK/LIMK and Inhibited PI3K/AKT/GSK-3β Pathways in BMECs

**Figure 3:**
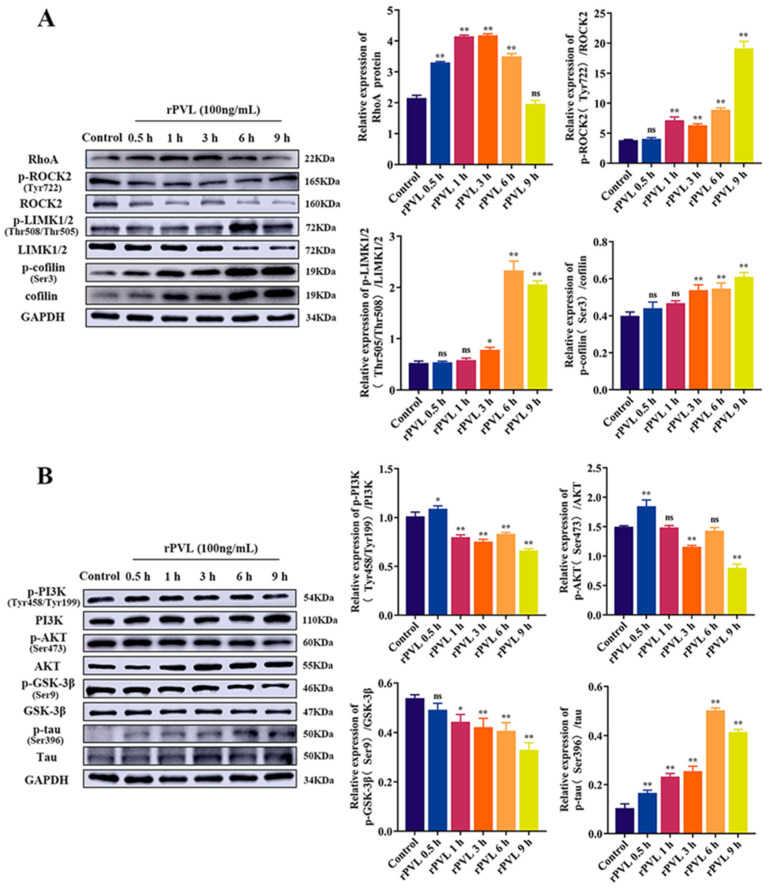
Effects of rPVL on the regulation of RhoA/ROCK/LIMK/Cofilin and PI3K/AKT/GSK-3β signaling pathways and phosphorylation of cofilin and tau hyperphosphorylation in the rPVL-treated BMECs. Representative immunoblot bands for RhoA, p-ROCK2(Tyr722), ROCK2, p-LIMK1/2(Thr508/Thr505), p-cofilin (Ser3), and cofilin (**A**); p-PI3K (Tyr458/Tyr199), PI3K, p-AKT(Ser473), AKT, GSK-3β(Ser9), GSK-3β, p-tau (Ser396), and tau (**B**); GAPDH was used as a control. rPVL was used at a 100 ng/mL concentration. Data are expressed as mean ± standard deviation of three independent experiments. * 0.01 < *p* < 0.05, ** *p* < 0.01 (one-way ANOVA with Dunnett’s multiple comparison tests), ns: not significant.

RhoA/ROCK/LIMK is a key signaling pathway for regulating the structure and function of the cytoskeleton. Additionally, the PI3K/AKT signaling pathway that induces phosphorylation of GSK-3β is also involved in cytoskeletal rearrangement. Therefore, we investigated whether the above pathways are involved in the rPVL-induced BMECs structural disorder. Western blot showed that the protein level of p-ROCK2(Tyr722)/ROCK2, p-LIMK1/2(Thr508/Thr505)/LIMK1/2, p-cofilin (Ser3)/cofilin (*p* < 0.05), and RhoA (*p* < 0.05) was significantly upregulated in the 100 ng/mL rPVL group compared to that of the controls at different points in time (Figure 3A). Moreover, the protein level of p-PI3K(Tyr458/Tyr199)/PI3K, p-AKT(Ser473)/AKT, and GSK-3β(Ser9)/GSK-3β was significantly downregulated (*p* < 0.05, Figure 3B), and p-tau (Ser396)/tau was significantly upregulated (*p* < 0.05, Figure 3B). These results indicated that the effects of rPVL on BMECs cytoskeleton remodeling depend on the RhoA/ROCK/LIMK and PI3K/AKT/GSK-3β pathway.

### 2.4. Baicalin Attenuated rPVL-Induced Rearrangement of Microfilaments and Microtubules in BMECs

**Figure 4:**
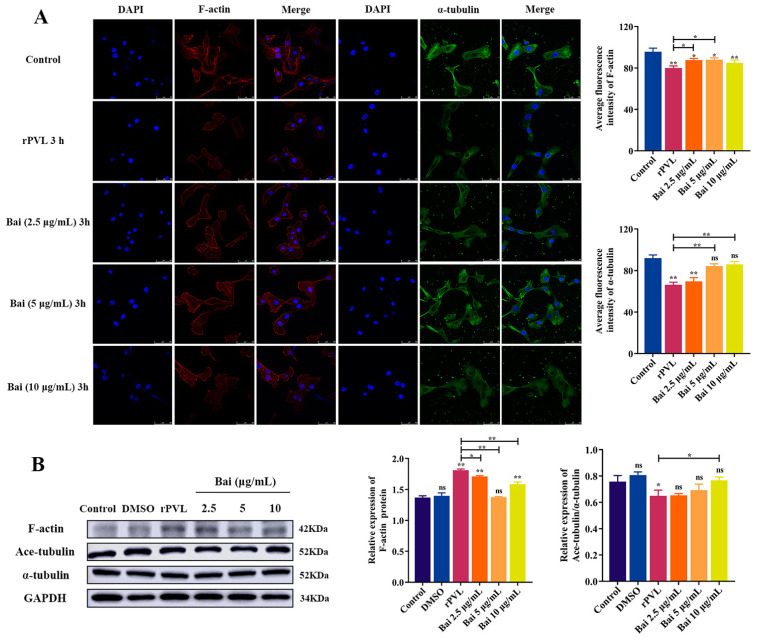
Effect of baicalin on the rearrangement of microfilaments and microtubules in rPVL-infected BMECs. (**A**) Effects of baicalin on the microfilaments and microtubules morphological damage in the rPVL-stimulated BMECs. Labeled with Rhodamine–Phalloidin (red), anti-α-tubulin antibody (green) and DAPI (blue). Scale bars are 50 μm in all figures. (**B**) Effects of baicalin on the abundance of F-actin, Ace-tubulin, and α-tubulin in rPVL-infected BMECs. Western blot was used to determine the relative F-actin, Ace-tubulin, and α-tubulin levels. GAPDH was used as a control. rPVL was used at a concentration of 100 ng/mL. Baicalin was used at 2.5, 5, and 10 μg/mL concentrations. Data are expressed as mean ± standard deviation of three independent experiments. * 0.01 < *p* < 0.05, ** *p* < 0.01 compared to the control, unless indicated with brackets (one-way ANOVA with Tukey’s multiple comparison tests), ns: not significant.

Some studies have shown the actin cytoskeleton regulation role of baicalin [34,35]. Therefore, we tested the role of baicalin on rPVL-induced BMEC rearrangement of microfilaments and microtubules. Laser confocal microscopy showed that pretreatment with baicalin was less rounded, and it restored microfilament (baicalin: 2.5 and 5 μg/mL) and microtubule fiber (baicalin: 5 and 10 μg/mL) structures in the PVL-treated BMECs compared to controls (with increased fluorescence intensity, *p* < 0.05, Figure 4A). Pretreatment with baicalin restored the protein abundance ratio of Ace-tubulin/α-tubulin (*p* < 0.05) and reduced the level of F-actin (*p* < 0.05, Figure 4B) in the PVL-treated BMECs. Thus, rPVL-induced damage to the cytoskeleton could be inhibited by baicalin treatment in BMECs.

### 2.5. Baicalin Attenuated the rPVL-Induced Rearrangement of Microfilaments and Microtubules in BMECs via the RhoA/ROCK/LIMK and PI3K/AKT/GSK-3β Signaling Pathways

**Figure 5:**
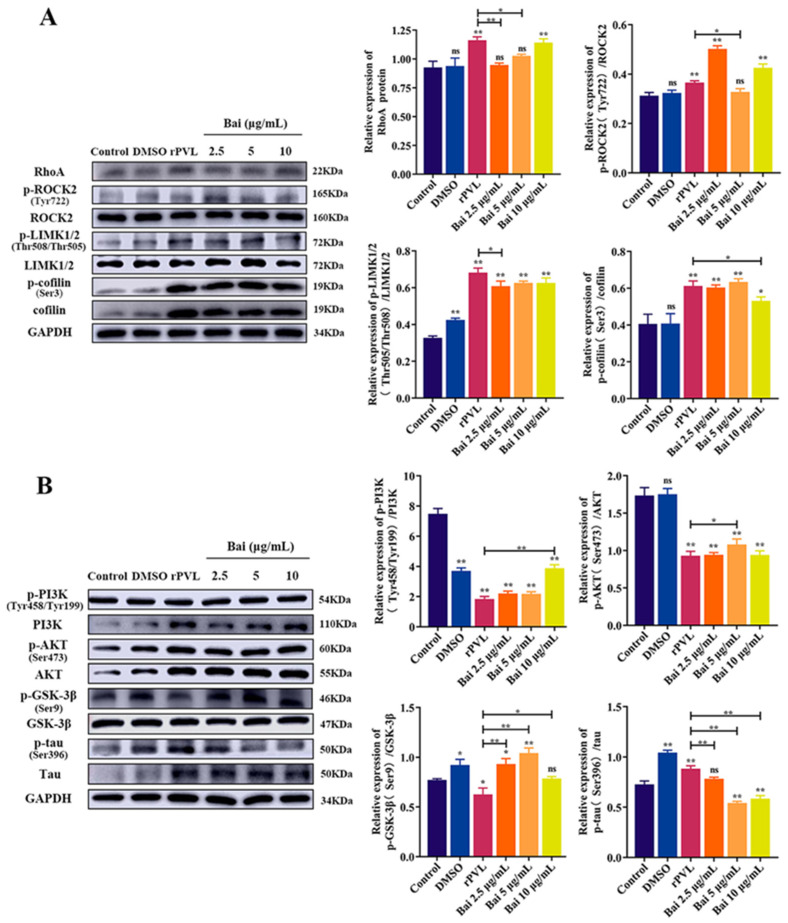
Baicalin attenuates rPVL-induced rearrangement of microfilaments and microtubules in BMECs via the RhoA/ROCK/LIMK and PI3K/AKT/GSK-3β pathways. Representative immunoblot bands for RhoA, p-ROCK(Tyr722), ROCK2, p-LIMK1/2(Thr508/Thr505), p-cofilin (Ser3), and cofilin (**A**); p-PI3K (Tyr458/Tyr199), PI3K, p-AKT(Ser473), AKT, GSK-3β(Ser9), GSK-3β, p-tau (Ser396), and tau (**B**); GAPDH was used as a control. rPVL was used at 100 ng/mL. Baicalin was used at concentrations of 2.5, 5, and 10 μg/mL. Data are expressed as mean ± standard deviation of three independent experiments. * 0.01 < *p* < 0.05, ** *p* < 0.01 compared to the control, unless indicated with brackets (one-way ANOVA with Tukey’s multiple comparison tests), ns: not significant.

We investigated the underlying regulatory mechanism of baicalin in the rPVL-induced rearrangement of microfilaments and microtubules. Pretreatment with baicalin reduced the protein level of p-ROCK2(Tyr722)/ROCK2 (baicalin: 5 μg/mL), p-LIMK1/2(Thr508/Thr505)/ LIMK1/2 (baicalin: 2.5 μg/mL), p-cofilin (Ser3)/cofilin (baicalin: 10 μg/mL) (*p* < 0.05) and RhoA (baicalin: 2.5 and 5 μg/mL) (*p* < 0.05) compared with those that were PVL-stimulated (Figure 5A). Pretreatment with baicalin restored the protein level of p-PI3K(Tyr458/Tyr199)/PI3K (baicalin: 10 μg/mL), p-AKT(Ser473)/AKT (baicalin: 5 μg/mL), and GSK-3β(Ser9)/GSK-3β (baicalin: 2.5, 5, and 10 μg/mL) (*p* < 0.05, Figure 3B). p-tau (Ser396)/tau (baicalin: 2.5, 5, and 10 μg/mL) was significantly downregulated (*p* < 0.05, Figure 5B).

## 3. Discussion

**Figure 6:**
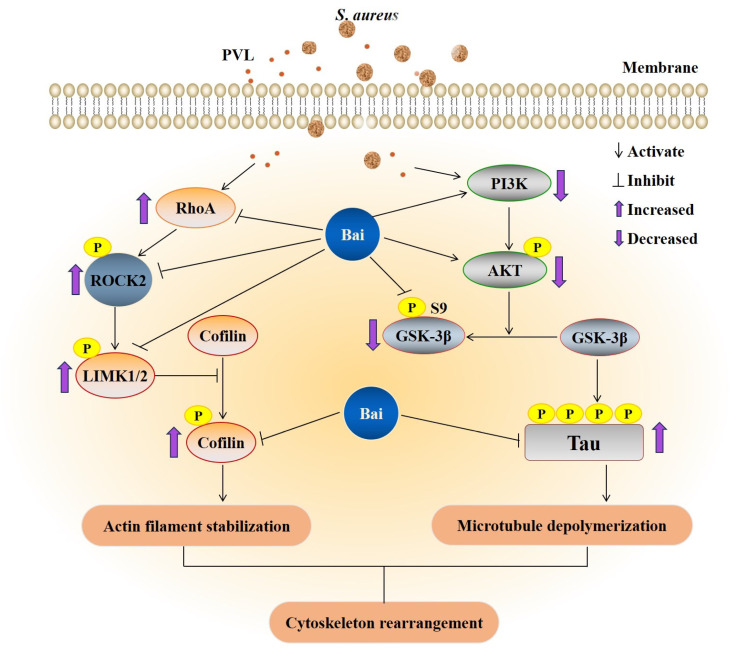
The schematic drawing illustrating the action mechanisms underlying the effects of baicalin on cytoskeleton rearrangement induced by PVL in BMECs. Baicalin inhibited cofilin phosphorylation and tau hyperphosphorylation by modulating the RhoA/ROCK/LIMK and PI3K/AKT/GSK-3β signaling pathways, reversing the destroying dynamic balance of actin and microtubule and recovering cellular structure. The purple arrows represent increased (upwards) or decreased (downwards) protein expression level in signaling pathway.

In this study, the pathogen (toxin)/host cell co-culture model we constructed using *S. aureus* and rPVL treatment validated that *S*. *aureus* induced cytoskeleton rearrangement mainly through PVL. rPVL exerted its cytoskeletal cytotoxicity via RhoA/ROCK/LIMK and PI3K/AKT/GSK-3β signaling activation, which caused phosphorylation of the cofilin and tau hyperphosphorylation, resulting in impairment of BMEC morphology and cell layer integrity. Baicalin attenuated cell shape alterations induced by PVL through regulation of the RhoA/ROCK/LIMK and of PI3K/AKT/GSK-3β pathways (Figure 6). These findings provide new insights into the pathogenesis and treatment of *S*. *aureus* infection in bovine mastitis.

Many bacterial pathogen product toxins rearrange the host cell cytoskeleton to promote infection [36]; bacterial toxins can directly affect structural proteins of the cytoskeleton (for example, the *V*. *cholerae* MARTX toxin and the binary actin-ADP-ribosylating toxins) or alter functions of cytoskeleton regulators (for example, the Rho-activating/inactivating bacterial toxins) [37,38]. The PFTs effects of *S*. *aureus*-derived Hla and EDIN on the cytoskeletal structures and its signal regulation have been investigated in different cell types [11,12,13]. One study demonstrated that PVL-induced neutrophil extracellular traps (NETs) are more enriched in cytoskeleton proteins such as actin, myosin, and tubulin than phorbol 12-myristate 13-acetate-induced NETs at the proteomic level [39], implying that PVL can induce neutrophil degradation of the cytoskeleton. In this study, a laser confocal microscope showed that treatment of BMECs with 100 ng/mL rPVL induced the instability and rearrangements of F-actin and tubulin cytoskeleton, and altered cell morphology. These phenomena are similar to the changes observed in intact *S*. *aureus*, suggesting PVL is responsible for the ability of *S*. *aureus* to induce cytoskeleton remodeling in BMECs. Such processes in early states of infection might limit the barrier function and might contribute to *S*. *aureus* invasion of BMECs.

Transition of actin cytoskeleton is tightly regulated in space and time by large quantities of signaling, scaffolding and actin-binding proteins [40]. The RhoA/ROCK/LIMK and PI3K/AKT/GSK-3β signaling pathway plays a crucial role in modulating actin assembly in various cellular types in response to extracellular stimuli [41]. Previous studies showed that *S*. *aureus* [9] and *S*. *agalactiae* [42] infection induced BMEC actin–cytoskeleton rearrangement through Rho GTPase (RhoA) regulated pathway modulation. The internalization of *S*. *aureus* and phosphorylation of GSK-3α (Ser21),and GSK-3β (Ser9) are associated with the PI3K/Akt signaling pathway in bovine endothelial cells [43]. In this study, Western blot revealed that PVL-mediated changes in cofilin phosphorylation at Ser3 require the activation of the RhoA/ROCK/LIMK signaling pathway in BMECs. PVL also triggered tau hyperphosphorylation at Ser396 and decreased the ratios of p-PI3K (Tyr458/Tyr199)/PI3K, p-Akt (Ser473)/Akt and p-GSK-3β (Ser9)/GSK-3β in BMECs, suggesting that both pathways induced the microfilament reorganization to change cell morphology.

Several recent studies have revealed that baicalin regulates cell functions via inhibition/activation of RhoA/ROCK [44,45,46] and PI3K/AKT/GSK-3β signaling pathways [47,48,49]. This study showed that 2.5, 5, and 10 μg/mL baicalin obviously ameliorates PVL-induced morphological changes in BMECs. The possible mechanism is that baicalin reduces cofilin phosphorylation at Ser3 and tau hyperphosphorylation at Ser396 by inhibiting the activation of RhoA/ROCK/LIMK and PI3K/AKT/GSK-3β signaling pathways. Toxin-induced apoptosis could be linked to cytoskeletal modifications [50,51], and baicalin could ameliorate PVL-induced BMECs apoptosis in vitro [32]. We suggest that baicalin inhibits rPVL-induced apoptosis by attenuating cytoskeletal rearrangements in BMECs. The effects of baicalin show positive results on pathogens causing mastitis and do not induce resistance after prolonged exposure [52,53,54]. Host cytoskeletal components are essential for bacterial lifecycles and evasion of host immune responses. Targeting bacteria/bacteria toxin–host cytoskeleton interactions by baicalin may provide novel approaches to *S*. *aureus*-induced bovine mastitis antibacterial treatment.

There are some limitations to this study. Bacteria toxins can activate RhoA through post-translational modification such as ADP-ribosylation, glucosylation, and proteolysis. These blocked interactions with ROCK can prevent the formation of actin stress fibers. In our study, the molecular mechanism of PVL-induced RhoA activation is not verified. The receptor for PVL on the surface of neutrophils is the C5a receptor; there is currently no evidence for the presence of C5aR on BMECs. Therefore, it is not clear whether rPVL causes cytoskeletal damage in BMECs by binding to cell surface receptors or by internalization via endocytosis. In addition, only in vitro test results were obtained here; they should be necessarily translated into in vivo settings and validate the effect of PVL-induced mammary gland injury and the dosage sufficiency or safety of baicalin.

## 4. Materials and Methods

### 4.1. Bacterial Strains and Cell Culture

*S*. *aureus* ATCC49775 producing PVL was purchased from the American Type Culture Collection (ATCC). The *pvl* gene knockout *S. aureus* strain Δ*pvl* 49775 and *pvl* gene-complemented *S. aureus* strain C-Δ*pvl* 49775 was gifted by Professor Wanjiang Zhang (Harbin Veterinary Research Institute, Harbin, China). The construction and growth characteristics of strains can be found in [55]. Bacteria were prepared by shaking (120 rpm) in tryptic soy agar (Haibo Ltd., Qingdao, China) broth overnight at 37 °C. For infections, *S*. *aureus* was grown to a mid-log phase and then collected by centrifugation, washed with sterile phosphate-buffered saline (PBS), and diluted to the required concentration (MOI = 30:1). BMECs stored by our laboratory were cultured in Dulbecco’s Modified Eagle’s Medium (DMEM) (Biological Industries, (BI), Kibbutz Beit-Haemek, Israel) supplemented with 10% fetal bovine serum (*v*/*v*) (Gibco, Waltham, MA, USA) and 100 U/mL penicillin–streptomycin (Sigma-Aldrich, St. Louis, MO, USA), and cultured at 37 °C in a humidified atmosphere with 5% CO_2_. Cells were cultured until they reached 80–90%.

### 4.2. Antibodies and Other Reagents

GSK-3β (Cat. No. bs-0023M) and F-actin antibodies (Cat. No. bs-1572R) were purchased from BIOSS (Beijing, China). The α-tubulin antibody (Cat. No. 66031-1-lg) was purchased from Proteintech (Rosemont, IL, USA). GAPDH (Cat. No. T0004), p-ROCK2(Tyr722, Cat. No. AF3028), acetyl-α-tubulin (Cat. No. AF4351), PI3K (Cat. No. AF5112), p-PI3K (Tyr458/Tyr199, Cat. No. AF3242), AKT (Cat. No. AF6259), p-AKT (Ser473, Cat. No. AF0016), p-GSK-3β (Ser9, Cat. No. AF2016), tau (Cat. No. AF6141), p-tau (Ser396, Cat. No. AF3148), RhoA (Cat. No. AF6352), ROCK2 (Cat. No. DF7466), LIMK1/2 (Cat. No. AF6344), p-LIMK1/2(Thr508/Thr505, Cat. No. AF3344), cofilin (Cat. No. AF6232), and p-cofilin (Ser3, Cat. No. AF3232) antibodies were purchased from Affinity (Liyang, China). Goat anti-rabbit IgG (ZB-2301) and goat anti-mouse IgG (SNP-9002) antibodies were purchased from Beijing Zhongshan Golden Bridge Biotechnology Co. Ltd. (Beijing, China). YF488 goat anti-mouse/rabbit IgG (Y6104), Rhodamine–Phalloidin (YP0063S) and 4′,6-diamidino-2-phenylindole (DAPI; D4080) were purchased from Suzhou Yuheng Biotechnology Co. Ltd. (Beijing, China). Baicalin standard substances (SB8020) and lysostaphin (L9070) were purchased from Beijing Solarbio Science and Technology Co., Ltd. (Beijing, China). Bicinchoninic acid protein quantitation assay (KGPBCA) and whole cell lysis assay (KGP2100) kits were purchased from Keygen Biotech (Nanjing, China).

### 4.3. rPVL Preparation

Expression and purification of rPVL have been described by Ma. et al. [56]. In summary, the *E*. *coli* BL21 strain was transformed with the pET28a-LukS-PV and pET28a-LukF-PV plasmids. LukS-PV and LukF-PV expressed by the transformed bacteria (LukS-PV^+^, LukF-PV^+^) strain were purified according to the His-Bind Purification Kit (Novagen, Darmstadt, Germany), and aliquoted into one-time-use stocks frozen at −80 °C.

### 4.4. Lysostaphin Protection Assays and In Vitro Inhibitor Treatment

BMECs were cultured at an initial density of 2 × 10^5^ cells per 6-well plates. For cell stimulation, BMECs were infected with *S*. *aureus* at an MOI of 30 or treated with 100 ng/mL rPVL according to [32]. At 3 h post infection, cells infected with *S*. *aureus* were washed three times with PBS, and incubated in a DMEM medium containing 10 μg/mL lysostaphin (Sigma-Aldrich, St. Louis, MO, USA) for 12 min. Subsequently, the medium was replaced with the DMEM medium to continue incubation. For baicalin intervention tests, BMECs were pretreated with 2.5, 5, and 10 μg/mL baicalin for 3 h, consecutively, and the cells were incubated with 100 ng/mL rPVL. At the indicated time points, the cell lysates were collected, purified, and analyzed using Western blot assays.

### 4.5. Immunofluorescence

Cells were cultured in a complete medium and treated at the indicated time as described above. The cells were washed thrice with PBS and fixed with 4% formaldehyde at 25 °C for 15 min. After permeabilization with 0.5% Triton X-100 for 10 min, and blocking with 5% bovine serum albumin for 1 h, cells were incubated with Rhodamine–Phalloidin for 15 min and an α-tubulin antibody for 2 h at 37 °C. Cells were then incubated with YF488 goat anti-rabbit IgG for 1 h at room temperature and stained with 4′,6-diamidino-2-phenylindole (DAPI) (5 µg/mL) for 10 min at room temperature. Finally, the specimens were mounted by anti-fluorescence quenching sealing tablets (Beijing, China) and observed using a laser confocal microscope (Leica, Wetzlar, Germany). ImageLab 6.1 software (BioRad, Hercules, CA, USA) was used for quantitation of fluorescence intensity values.

### 4.6. Western Blot Analysis

BMECs were washed thrice with PBS and lysed in cold radioimmunoprecipitation assay lysis buffer containing a protease inhibitor cocktail (P8340, Sigma-Aldrich, St. Louis, MO, USA) on ice for 30 min. Lysates were centrifuged at 12,000× *g* for 15 min. Protein concentration in the supernatant was measured by the Bradford assay method. Proteins were then boiled for 10 min and analyzed by sodium dodecyl sulfate-polyacrylamide gel electrophoresis (SDS-PAGE). For gel electrophoresis, 15 µg of total protein extracts was separated on 12.5% SDS-PAGE gel and then transferred to an Immuno-Blot polyvinylidene fluoride (Millipore, Temecula, CA, USA) membrane through wet transfer apparatus (BioRad, Hercules, CA, USA) for 1.5 h, at a 300 mA constant current. The membrane was incubated for 1 h with 5% non-fat dairy milk, washed thrice for 10 min, and the primary antibodies were probed overnight at 4 °C. The membranes were washed and probed with horseradish peroxidase (HRP) goat anti-rabbit IgG antibody and HRP goat anti-mouse IgG antibody for 1 h at 25 °C. Thereafter, chromogenic-enhanced chemiluminescence was performed. An AI600RGB imager (GE, Fairfield, CT, USA) and ImageLab 6.1 software (BioRad, Hercules, CA, USA) were used to reveal and analyze the chemiluminescence signal.

### 4.7. Statistical Analysis

Data were analyzed and graphed using GraphPad Prism 8.0 (GraphPad Software, San Diego, CA, USA). Statistical data are presented as means ± standard deviations of three independent experiments. One-way ANOVA with Dunnett’s multiple comparison tests was used to compare means from experimental groups against control group mean, or the data among subgroups were compared in pairs using StudentNewmanKeuls (SNK) tests. Significance levels were set at * *p* < 0.05 and ** *p* < 0.01, ns: not significant.

## 5. Conclusions

This study indicated that PVL activity of *S. aureus* is related with cytoskeleton rearrangement in BMECs. rPVL regulates the RhoA/ROCK/LIMK and PI3K/AKT/GSK-3β signaling pathways, thus effecting BMECs morphology, microfilament and the microtubule rearrangement. Baicalin attenuates cytoskeleton rearrangement in BMECs via modulating the activation of the RhoA/ROCK/LIMK and PI3K/AKT/GSK-3β signaling pathway. These findings provide new insights into the mechanisms and potential treatment of *S*. *aureus* causing bovine mastitis.

## Data Availability

The original contributions presented in the study are included in the article.
